# Novel Directed Enzyme Prodrug Therapy for Cancer Treatment Based on 2′-Deoxyribosyltransferase-Conjugated Magnetic Nanoparticles

**DOI:** 10.3390/biom14080894

**Published:** 2024-07-24

**Authors:** Elena Pérez, Javier Acosta, Victor Pisabarro, Marco Cordani, José C. S. dos Santos, Jon Sanz-Landaluze, Juan Gallo, Manuel Bañobre-López, Jesús Fernández-Lucas

**Affiliations:** 1 Applied Biotechnology Group, Universidad Europea de Madrid, Urbanización El Bosque, 28670 Villaviciosa de Odón, Spain; elena.perez2@universidadeuropea.es (E.P.); javier.acosta@universidadeuropea.es (J.A.); victorpisabarromontoro@gmail.com (V.P.); 2 Instituto de Investigaciones Sanitarias San Carlos (IdISSC), 28040 Madrid, Spain; 3 Department of Biochemistry and Molecular Biology, Faculty of Biology, Universidad Complutense de Madrid, C. de José Antonio Novais, 12, 28040 Madrid, Spain; mcordani@ucm.es; 4 Instituto de Engenharias e Desenvolvimento Sustentável, Universidade da Integração Internacional da Lusofonia Afro-Brasileira, Campus das Auroras, Redenção 62790970, CE, Brazil; jcs@unilab.edu.br; 5 Department of Analytical Chemistry, Faculty of Chemical Science, Universidad Complutense de Madrid, Avenida Complutense S/N, 28040 Madrid, Spain; jsanzlan@quim.ucm.es; 6 INL—International Iberian Nanotechnology Laboratory, Avenida Mestre José Veiga, 4715-330 Braga, Portugal; juan.gallo@inl.int (J.G.); manuel.banobre@inl.int (M.B.-L.); 7 Grupo de Investigación en Ciencias Naturales y Exactas—GICNEX, Universidad de la Costa, CUC, Calle 58 # 55–66, Barranquilla 080002, Colombia

**Keywords:** chemotherapy, selectivity, nucleoside analogs, 2′-deoxyribosyltransferase, enzyme immobilization, prodrug activation

## Abstract

Directed enzyme prodrug therapy (DEPT) strategies show promise in mitigating chemotherapy side effects during cancer treatment. Among these, the use of immobilized enzymes on solid matrices as prodrug activating agents (IDEPT) presents a compelling delivery strategy, offering enhanced tumor targeting and reduced toxicity. Herein, we report a novel IDEPT strategy by employing a His-tagged *Leishmania mexicana* type I 2′-deoxyribosyltransferase (His-*Lm*PDT) covalently attached to glutaraldehyde-activated magnetic iron oxide nanoparticles (MIONPs). Among the resulting derivatives, PDT-MIONP3 displayed the most favorable catalyst load/retained activity ratio, prompting its selection for further investigation. Substrate specificity studies demonstrated that PDT-MIONP3 effectively hydrolyzed a diverse array of 6-oxo and/or 6-amino purine 2′-deoxynucleosides, including 2-fluoro-2′-deoxyadenosine (dFAdo) and 6-methylpurine-2′-deoxyribose (d6MetPRib), both well-known prodrugs commonly used in DEPT. The biophysical characterization of both MIONPs and PDT-MIONPs was conducted by TEM, DLS, and single particle ICPMS techniques, showing an ideal nanosized range and a zeta potential value of −47.9 mV and −78.2 mV for MIONPs and PDT-MIONPs, respectively. The intracellular uptake of MIONPs and PDT-MIONPs was also determined by TEM and single particle ICPMS on HeLa cancer cell lines and NIH3T3 normal cell lines, showing a higher intracellular uptake in tumor cells. Finally, the selectivity of the PDT-MIONP/dFAdo IDEPT system was tested on HeLa cells (24 h, 10 µM dFAdo), resulting in a significant reduction in tumoral cell survival (11% of viability). Based on the experimental results, PDT-MIONP/dFAdo presents a novel and alternative IDEPT strategy, providing a promising avenue for cancer treatment.

## 1. Introduction

Cancer is a major public health concern, which, according to World Health Organization [1] (WHO 2020), caused around 9.6 million deaths and affected around 18.1 million new patients in 2018. These statistics highlight cancer as a leading cause of one in six deaths worldwide. Despite advancements in cancer treatment, projections suggest that by 2040, the disease will affect over 29.4 million new patients [1]. One of the earliest cancer treatment approaches involved the use of nucleoside analogs as anticancer agents. As of 2013, the Food and Drug Administration (FDA) approved up to 13 purine and pyrimidine antimetabolites as chemotherapeutic agents for cancer treatment [2]. Nevertheless, the clinical application of nucleoside analogs in cancer treatment faces various limitations, encompassing inadequate drug concentrations within tumors, systemic toxicity, a lack of selectivity for tumor cells, and the emergence of drug-resistant tumor cells [3,4].

Enzyme Prodrug Therapy (EPT), particularly Directed-Enzyme Prodrug Therapy (DEPT), aims to mitigate undesirable effects associated with conventional cancer treatment effects [5]. DEPT involves the selective activation of non-toxic prodrugs within tumor cells or the tumor microenvironment by exogenous enzymes [3,6,7]. This targeted activation results in elevated local concentrations of the toxic drug, thereby reducing systemic toxicity. However, to obtain a suitable application, DEPT strategies must address key considerations: (a) the prodrug must remain inert to endogenous human enzymes in non-tumor tissues; (b) exogenous proteins require moderate concentrations in tumor cells alongside high catalytic activity; (c) the prodrug and toxic drug must cross cell membranes for effective action, inducing a “bystander effect” in neighboring non-expressing enzyme tumor cells; (d) the toxic metabolite half-life should be long enough for a bystander effect in adjacent cells, but short enough to prevent systemic circulation leakage [5].

Achieving a sufficient concentration of exogenous enzymes within tumor tissues is a critical challenge in DEPT. In this context, two different DEPT strategies can be distinguished: (i) the delivery of genes encoding prodrug-activating enzymes into tumor cells (gene-directed enzyme prodrug therapy, GDEPT), employing virus or non-virus carriers [3,6,7], and (ii) the delivery of prodrug-activating enzymes into tumor cells using various carriers, such as tumor-associated monoclonal antibodies linked to prodrug-activating enzymes (antibody-directed enzyme prodrug therapy, ADEPT) [8] or the solid matrixes (immobilized-directed enzyme prodrug therapy, IDEPT) [9,10].

Due to their ability to convert nucleoside prodrugs into active cytotoxic agents specifically within cancer cells, the utilization of enzymes from purine and pyrimidine salvage pathways in DEPT strategies is gaining ground in contemporary research for cancer treatment [11]. By harnessing salvage pathway enzymes, prodrugs can be selectively activated in tumor cells, minimizing off-target effects, and reducing systemic toxicity. Additionally, enzymes from purine and pyrimidine salvage pathways offer a diverse range of substrates and activation mechanisms [12,13,14], allowing for the development of tailored prodrug/enzyme combinations to target different types of cancer or even specific molecular subtypes within a cancer type. This versatility enhances the precision and efficacy of EPT strategies, making them promising candidates for cancer therapy.

In this context, the use of purine and pyrimidine salvage enzymes in DEPT therapies for cancer treatment has been extensively reported, with a predominant focus on GDEPT [5]. Among them, we must highlight enzyme/prodrug systems based on the use of purine and pyrimidine salvage enzymes [11], such as bacterial cytosine deaminase/5-fluorocytosine (CytDA/FCyt) [15], thymidine phosphorylase/5-fluorouracil (TP/5-FUra) or 5′-deoxyfluorouridine (TP/5′-dFUra) [16], *E. coli* purine nucleoside phosphorylase/Fludarabine (*Ec*PNP/Fludarabine) [17,18,19], or the recently reported system *Lactobacillus delbrueckii* type II 2′-deoxyribosyltransferase/2′-deoxy-2-fluoroadenosine (*Ld*NDT/dFAdo) [6]. However, gene delivery strategies confront challenges such as low efficiency in the transduction process, protein expression issues, insertional mutagenesis, or the risk of infections, among others [5,7,8]. These drawbacks can be overcome by ADEPT and IDEPT strategies. Still, they present additional complications, including selectively targeting tumoral cells and the difficulty of large antibody-enzyme conjugates or immobilized enzyme derivates to diffuse across cell membranes.

Much of the credit for the rise of IDEPT strategies relies upon the advantages associated with enzyme immobilization. These benefits encompass enhanced stability and shelf life, paving the way for targeted delivery of prodrug-activating enzymes specifically to tumor sites. Matrices like colloidal gold and magnetic nanoparticles have been explored for IDEPT strategies, with magnetic iron oxide nanoparticles (MIONPs) showing promise due to their magnetic responsiveness and biocompatibility [10]. MIONPs enable magnetic targeting and monitoring through magnetic resonance (MR). Additionally, the enhanced permeability and retention (EPR) effect in tumors, attributed to altered blood vessel architecture and increased vascular permeability factors, further promotes the accumulation of nanoparticles in cancerous tissues, selectively delivering macromolecules larger than 40 kDa and aiding in the tumor’s nutrient supply for rapid growth. This phenomenon is notably absent in healthy tissues, where vascular permeability is significantly lower [20].

While numerous examples of enzyme/prodrug systems based on IDEPT strategies have been developed in the recent decades [10,21,22,23,24], there has been a notable absence of efforts focused on utilizing purine and pyrimidine salvage enzymes in IDEPT, with the sole exception being those based on cytosine deaminase/prodrug combinations [9,25].

**Figure 1:**
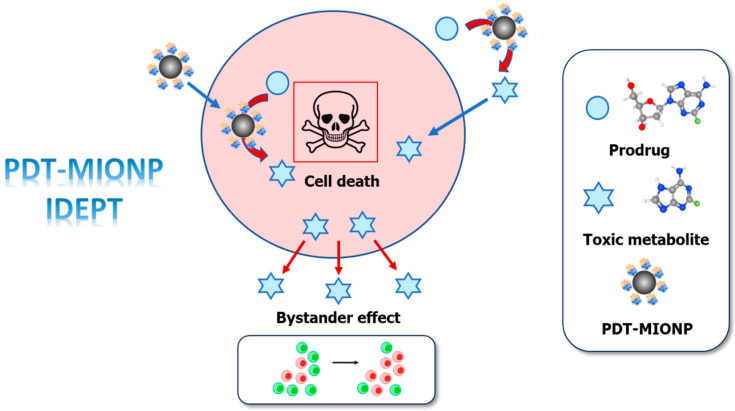
General scheme of PDT-MIONP/dFAdo IDEPT approach.

Building on our prior findings in the GDEPT system *Lactobacillus delbrueckii* type II 2′-deoxyribosyltransferase/2′-deoxy-2-fluoroadenosine (*Ld*NDT/dFAdo) [6,26], but also on enzyme immobilization [27,28], we envisioned an IDEPT strategy based on the activation of nucleoside prodrugs through immobilized 2′-deoxyribosyltransferases onto MIONPs (Figure 1) (Figure S1).

Herein, we introduce a novel IDEPT approach using His-tagged *Leishmania mexicana* type I 2′-deoxyribosyltransferase (His-*Lm*PDT) immobilized on glutaraldehyde-activated magnetic iron oxide nanoparticles (MIONPs). PDT-MIONP3 emerged as the most promising derivative, exhibiting effective hydrolysis of various purine 2′-deoxynucleosides, including 2-fluoro-2′-deoxyadenosine (dFAdo) and 6-methylpurine-2′-deoxyribose (d6MetPRib) (Figure S1), common prodrugs in DEPT. Biophysical characterization confirmed nanoscale dimensions and zeta potentials for both MIONPs and PDT-MIONPs, with enhanced intracellular uptake observed in tumor cells. Finally, the evaluation of the PDT-MIONP/dFAdo IDEPT system on HeLa cells demonstrated a significant reduction in tumoral cell survival (11% viability).

## 2. Materials and Methods

### 2.1. Materials

The cell culture medium reagents were sourced from Difco (St. Louis, MO, USA). Triethyl ammonium acetate buffer was obtained from Sigma-Aldrich in Madrid, Spain. All additional reagents and organic solvents were procured from Symta (Madrid, Spain). All the nucleosides and nucleobases utilized in this study were supplied by Carbosynth Ltd. (Compton, UK). 

### 2.2. Enzyme Production and Purification

The recombinant His-*Lm*PDT was produced and purified following well-established protocols [29]. Protein concentration was determined using UV_280_ nm spectroscopy, with a molar extinction coefficient of 8940 M^−1^ cm^−1^ derived from the amino acid sequence.

### 2.3. Standard Enzymatic Activity Assay

The protocol utilized for assessing the glycosidase activity of the soluble enzyme was adapted from established methodologies outlined in the previous literature [6,17,19]. Specifically, the enzyme-mediated glycosidase assay was conducted by adding 0.3 μg of pure His-*Lm*PDT into the reaction mixture, consisting of 5 mM 2′-deoxyinosine (dIno) in 50 mM PBS buffer at pH 7.4, maintained for 5 min at 37.5 °C. Subsequently, the enzymatic reaction was terminated by the addition of 40 μL of cold methanol in an ice bath, followed by heating for 5 min at 95 °C. Upon centrifugation at 8500× *g* for 5 min, the resulting samples were half-diluted with water, and the presence of hypoxanthine (Hyp) was analyzed and quantified through HPLC. Under these specified conditions, one unit of activity (U) was defined as the quantity of enzyme (mg) yielding one µmol min^−1^ (IU) of Hyp under the assay conditions.

### 2.4. Preparation and Functionalization of MIONPs 

The synthesis of oleic acid-stabilized magnetite nanoparticles was prepared following a protocol based on Ref. [30]. To this end, 0.5 g (1.6 mmol) of sodium oleate was dissolved in 5 mL of water with the help of a sonicating bath. Separately, the iron salts (FeCl_2_.4H_2_O, 1.59 g, 8 mmol; FeCl_3_.6H_2_O, 3.78 g, 14 mmol) were mixed and dissolved in 7 mL of water. Both solutions were mixed in a 40 mL Teflon reactor, to which 15 mL of ammonia (28% *v*/*v*) was added. The reactor was sealed, placed in a stainless steel autoclave, and heated at 200 °C for 36 h. After cooling to room temperature, the black slurry product was washed with water, centrifugated (9000 rpm, 5 min) three times, and dried under vacuum over 72 h. Following the grinding of the dried product, 50 mL of hexane was added for solubilizing the MIONPs. After sonication for 30 min, 150 mL of isopropanol was added to precipitate the nanoparticles (NPs). The NPs were collected by centrifugation (9000 rpm, 5 min), and the process was repeated once. The pellet was resuspended in hexane (10 mL) and centrifuged at 3000 rpm for 10 min to remove large aggregates. The supernatant was stored at 4 °C until further use.

The functionalization of the previously prepared nanoparticles was conducted following a protocol outlined in [31]. Initially, 1 mL of the hexane Fe_3_O_4_ nanoparticle solution underwent a pre-cleaning process, involving three cycles of removal from solution using acetone and subsequent resuspension in hexane. This resulting suspension was then diluted to 30 mL with hexane. Subsequently, 10 μL of acetic acid was introduced into the partially cloudy solution to catalyze silanization. Following this, 450 μL of (3-Aminopropyl) triethoxysilane (APTES) was added, resulting in a complete clarification of the solution. The reaction proceeded in an Erlenmeyer flask placed on an orbital shaker at 21 °C for 48 h. Upon completion, the sample precipitated entirely. It was then resuspended in a sonication bath and separated using a handheld magnet. The resulting pellet underwent washing with MeOH followed by magnetic separation. Water was subsequently added to the pellet, yielding a clear solution that gradually flocculated and could be sorted magnetically. This solution was further resuspended in MeOH to achieve stability. Following purification with a magnet over the weekend, the solution was resuspended in water and ultimately diluted to a concentration of 0.5 mg Fe mL^−1^, as determined by ICP-OES measurements.

### 2.5. Enzyme Immobilization 

The immobilization of His-*Lm*PDT was achieved through the covalent bonding of the enzyme onto glutaraldehyde-activated MIONPs [28,32]. For this purpose, 100 µL of nanoparticle suspension (914 µg mL^−1^) was washed and equilibrated with activation buffer (50 mM sodium phosphate pH 8.5). MIONPs were collected by a magnetic separator and the supernatant was discarded. Support activation was performed by incubating the nanoparticles with 150 µL of 50 mM sodium phosphate buffer pH 8.5, containing 1 M glutaraldehyde (9.33% *w*/*v*) for 3 h at room temperature (RT) in a roller shaker. 

After that, the activated nanoparticles were thoroughly washed and equilibrated with a binding buffer (50 mM sodium phosphate pH 7.5) to eliminate excess glutaraldehyde. Afterward, 10–40 µg of the enzyme was mixed with the activated nanoparticles and binding buffer, and the final volume was adjusted to 50 µL. Then, the enzyme/nanoparticle suspension was incubated at RT for 20 h in a roller shaker.

Subsequently, nanoparticles were collected by a magnetic separator and the supernatant was kept for posterior analysis. Nanoparticles were rinsed with washing buffer (50 mM sodium phosphate pH 8.5) and treated with glycine 3 N for 3 h at room temperature to remove the non-covalently bound enzyme and quench any remaining amine reactive residues in the support. Finally, the nanoparticles were washed with 50 mM sodium phosphate buffer pH 8 and stored at 4 °C. 

### 2.6. Standard Enzymatic Activity Assay for the Immobilized His-LmPDT

Similarly to soluble enzymes, the enzymatic activity of immobilized derivatives (PDT-MIONPs) was assessed using dIno as a substrate. The assay was conducted by adding 0.6 µg of immobilized (PDT-MIONP1-3) or free enzyme (His-*Lm*PDT) to a solution containing 1 mM dIno and phosphate-buffered saline (PBS) buffer (1×, pH 7.4) in a final volume of 80 µL. This reaction mixture was then incubated for 5 min at 40 °C and 300 rpm. The reaction was quenched by collecting the nanoparticles with a magnetic separator, half-diluting the supernatant with cold methanol, and incubating it in an ice bath for 5 min. Subsequently, another 5 min incubation at 95 °C was performed. Finally, the samples were centrifuged at 9000× *g* and half-diluted with water. Quantitative analysis of Hyp formation was conducted by HPLC analysis, as described below. Again, all determinations were performed in duplicate, and the maximum error was less than 5%. One activity unit (U) was defined as the amount of immobilized derivative (grams of support, g_sup_) producing one µmol min^−1^ (IU) of hypoxanthine under the assay conditions.

### 2.7. Substrate Specificity of PDT-MIONP Derivative

To evaluate the glycosidase activity of the immobilized enzyme, 2.6 µg of PDT-MIONP3 (comprising 0.6 µg of immobilized enzyme) was introduced into a mixture containing 1 mM 2′-deoxynucleoside (dIno, dGuo, dAdo, 2-FdAdo, and d6MetPRib) (Figure S1) and PBS (1×, pH 7.4). This mixture was incubated at 40 °C for 5 min and 300 rpm, in a final volume of 80 µL. The reaction was stopped as previously described for the standard activity assay and the glycosidase activity was quantitatively determined by HPLC analysis, as described in the Analytical Methods section.

### 2.8. Analytical Methods

The glycosidase activity of both soluble and immobilized enzyme was analyzed by HPLC using an ACE EXCEL 5 µm CN-ES column 250 mm × 4.6 mm equilibrated and eluted with 100% Milli-Q^®^ water (deionized water) at a flow rate of 0.8 mL min^−1^. The diode array detector for quantification was fixed at 254 nm. Retention times for the reference compounds (from here on, abbreviated according to the recommendations of the IUPAC-IUB Commission on Biochemical Nomenclature) were the following: adenine (Ade), 9.9 min; guanine (Gua); 5.4 min; hypoxanthine (Hyp), 5.1 min; 2-fluoroadenine (2-FAde), 12.2 min; 6-methylpurine (6-MetPur), 12.3 min; 2′-deoxyadenosine (dAdo), 18.9 min; 2′-deoxyguanosine (dGuo), 9.1 min; 2′-deoxyinosine (dIno), 7.4 min; 6-methylpurine-2′-deoxyribose (d6MetPRib), 27.1 min; and 2′-deoxy-2-fluoroadenosine (dFAdo), 27.0 min (Figure S1).

### 2.9. Cell Lines and Cell Culture

Human cervical cancer cells (HeLa) and embryonic mouse fibroblasts (NIH3T3) were obtained from the European Collection of Authenticated Cell Cultures, ECACC (Porton Down, Wiltshire, England) and grown in Dulbecco’s Modified Eagle Medium (DMEM) + GlutaMax^TM^ supplemented with 10% heat-inactivated fetal bovine serum, penicillin (50 U mL^−1^), and streptomycin (50 µg mL^−1^) [6]. Cells were maintained in a humified incubator HERAcell CO_2_ (Thermo Fisher Scientific, Madrid, Spain) at 37°C and 5% CO_2_ atmosphere. For further experiments, cells were grown to confluence as monolayers, trypsinized (0.05% trypsin/0.53 mM EDTA), and plated. The absence of mycoplasma and other contaminants in cell cultures was regularly checked.

### 2.10. Biophysical Characterization 

Biophysical characterization of both MIONPs and PDT-MIONPs was conducted via transmission electron microscopy (TEM), dynamic light scattering (DLS), and single particle inductively coupled plasma mass spectrometry (ICP-MS) techniques.

In this context, the determination of nanoparticle size and morphology was determined using transmission electron microscopy (TEM). The JEOL JEM 2100 unit was operated at an accelerating potential of 80–200 kV (International Iberian Nanotechnology Laboratory, Braga, Portugal). Moreover, the hydrodynamic size and zeta potential of MIONPs and PDT-MIONPs were also analyzed by DLS Horiba SZ-100Z (International Iberian Nanotechnology Laboratory, Braga, Portugal) after diluting both nanosystems in Milli-Q^®^ water. Additionally, those parameters were also determined before and after 5 min of sonication to analyze the aggregation behavior of MIONPs in solution. Finally, the size of MIONPs and PDT-MIONPs in culture media was determined by single particle ICP-MS as described below.

### 2.11. Cytotoxicity Analysis

#### Cell Viability Assays Using MIONPs and PDT-MIONPs

HeLa cells were seeded at a rate of 5 × 10^3^ cells per well in 96-well plates and incubated overnight. After that, 100 µL of different concentrations of MIONPs (0.01–0.1 mg mL^−1^) or immobilized derivates (PDT-MIONPs, 0.01–0.1 mg mL^−1^) were added to cells and incubated for 24 h. To study the effect of nanoparticle aggregation, the toxicity of MIONPs was also assessed with and without sonication in an ultrasonic bath for 30 min before their addition to cell cultures. In the case of PDT-MIONPs, their effect on cell viability was only assayed without sonication, as this procedure may affect negatively to enzyme immobilization and folding. In all cases, mitochondrial activity was determined by the MTT tetrazolium compound [3-(4,5-dimethlthiazol-2-yl)-5-(3-carcoxymethoxyphenyl)-2-(4-sulfophenyl)-2H-tetrazolium] method, as previously described [6]. After 2 h, the excess of MTT was removed and 100 µL of dimethyl sulfoxide (DMSO) was added to dissolve the resulting formazan crystals, which are directly proportional to the number of viable cells in the culture. Afterwards, absorbance at 570 nm was measured using a standard microplate reader. Thus, relative cell viability (%) was determined in relation to control wells containing cell culture medium without nanoparticles according to the Equation (1),

([*A*]*_sample_*/[*A*]*_control_*) × 100 (1)

where [*A*]*_sample_* and [*A*]*_control_* correspond to the absorbance reads at 570 nm for each sample and control well, respectively.

### 2.12. Intracellular Uptake of MIONPs and PDT-MIONPs

The intracellular loading of MIONPs and PDT-MIONPs was determined by TEM and ICP-MS.

For TEM analysis, HeLa cells were seeded in 6-well plates at a density of 1.5 × 10^5^ cells per well and grown overnight. After that, 100 µL of 0.05 mg mL^−1^ PDT-MIONPs was added, and cells were incubated for 24 h at 37 °C. Then, cells were trypsinized, rinsed in a final volume of 1.5 mL of PBS, and collected by 5 min of centrifugation at 1500 rpm. Cells were fixed with Karnovsky’s fixative solution (paraformaldehyde 4% and glutaraldehyde 2.5% in phosphate buffer Millonig, 0.1 M, pH 7.3) for 4 h at 4 °C. After washing them 4 times with Millonig buffer, fixed cells were kept at 4 °C overnight. Subsequently, the samples were stained with 1% osmium tetroxide for 1 h and then washed with Milli-Q^®^ 3 times. Cell pellets were dehydrated with a series of acetone concentrations (30%, 50%, 70%, 80%, 90%, 95%, and 100%). Afterward, the resulting pellets were infiltrated in 1:3, 1:1, and 3:1 SPURR resin/acetone mixture for 1, 1, and 1.5 h, respectively. Finally, they were infiltrated in pure resin and kept overnight at room temperature. Subsequently, cell blocks were transferred to fresh resin and incubated at 65 °C for 48 h. Ultrathin cuts were obtained using a Leica Ultracut UCT and viewed under JEOL JEM 1400 TEM, operated with an acceleration potential of 40–120 kV (Centro Nacional de Microscopía Electrónica, Madrid, Spain).

For the ICP-MS study, the authors implemented a protocol based on previously described methods [33,34]. In brief, HeLa cells at a density of 1.5 × 10^5^ cells/well and NIH3T3 cells at a density of 3 × 10^5^ cells/well were cultured in 6-well plates at 37 °C and 5% CO_2_ in Dulbecco’s Modified Eagle Medium (DMEM) supplemented with GlutaMaxTM and 10% heat-inactivated fetal bovine serum, along with penicillin (50 U mL^−1^) and streptomycin (50 µg mL^−1^). Upon reaching 70% confluence, the exponentially growing cells were treated by adding MIONPs at concentrations ranging from 50 to 100 µg mL^−1^. After 24 h, cell pellets and the surrounding growth media were collected for subsequent ICP-MS analysis. This analysis included total iron quantification in the cell pellets and nanoparticle size analysis in the growth media, utilizing the single particle mode of ICP-MS analysis. Cell pellets were washed with PBS (×3), counted, and lysed using an acid digestion protocol. This involved adding 0.5 mL of sub-boiling HNO_3_ and 0.5 mL H_2_O_2_ (30%) and incubating at 70 °C for 4 h. The growing media were measured without any additional treatment. For ICP-MS quantification, an Agilent 7700× instrument from Agilent Technologies (Santa Clara, CA, USA) was utilized, equipped with an octopole reaction system (ORS) employing H_2_ as the reaction gas. The instrument was fitted with a concentric nebulizer, and hydrogen was utilized as the reaction gas to eliminate ^40^Ar^16^O and ^40^Ar^16^O^1^H polyatomic interferences affecting ^56^Fe and ^57^Fe, respectively. Calibration curves were generated using inorganic iron (Iron Standard for ICP, 1000 ± 2 mg L^−1^ Fe in 2% nitric acid, Sigma-Aldrich, St. Louis, MO, USA) for total determination in cells and MIONPs (50 µg/mL) for total determination in non-treated growing media. The mean of four separate replicates was used for the determination of iron concentration.

For the single particle configuration of ICP-MS, a MicroMist nebulizer combined with a total consumption spray chamber (High Sensitivity Single-Cell Sample Introduction System for ICP-MS, Glass Expansion, Port Melbourne, Australia) was used [35]. Samples and standards were introduced into the plasma at a flow rate of 10 μL min^−1^ using an Infusion Syringe Pump from FisherScientific. Measurements were conducted in time-resolved analysis (TRA) mode for 3 min for each sample. Transport efficiency was initially calculated using the particle frequency method employing citrate-gold nanoparticles (citrate-AuNPs) reference material LGC5050, with a nominal diameter of 32.7 ± 2 nm, obtained from LGC (Teddington, UK), diluted to a concentration of 40 ng/L. A quantification curve of ionic iron was prepared daily with concentrations ranging from 0.25 to 50 μg L^−1^ from an iron standard of 1000 mg L^−1^. Data analysis for sp-ICP-MS was performed using an iterative procedure previously described [36,37].

### 2.13. Application of PDT/Prodrug System on Cancer Cells

As a preliminary study, the effectiveness of this IDEPT strategy was evaluated in cell cultures. For this purpose, 5 × 10^3^ HeLa cells per well were seeded in 96-well plates and incubated overnight. Subsequently, 100 µL of 0.01 mg mL^−1^ and 0.05 mg mL^−1^ of PDT-MIONPs were added. Cells were then incubated at 37 °C for 5 h to facilitate intracellular internalization, after which residual nanoparticles not internalized were removed. Following this, different concentrations of the selected prodrug FdAdo (1, 2.5, and 10 µM) were added, and the NDT/Prodrug system was further incubated for 24 h. Finally, the relative cell viability (%) was determined using the MTT assay as previously described [6]. All conditions were performed in quintuplicate.

### 2.14. Statistical Analysis 

Statistical significance was assessed using an unpaired *t*-test two-tailed (*p* < 0.001) using Prism software version 8.0, GraphPad. All experiments were carried out at least in quintuplicate (n = 5) and the results were calculated as mean ± standard deviation (SD) as reported in the figure legend in each case.

## 3. Results and Discussion

### 3.1. Covalent Immobilization of His-LmPDT onto MIONPs

According to previous experimental procedures [29], His-*Lm*PDT was produced and purified through affinity chromatography. The presence of His-*Lm*PDT in the eluted fractions was confirmed by SDS-PAGE (Figure S2). Once His_-_*Lm*PDT was produced, we proceeded to determine the proper conditions for the immobilization process. Based on previous findings on the immobilization of NDTs [28,29,32,38,39], initially, we consider a uni-punctual covalent immobilization through a deprotonated N-terminus. To fulfill this objective, we constructed a 3D model of His-*Lm*PDT, encompassing the initial 19 amino acids at the N-terminus, leveraging the crystallized structure of dimeric *Lm*PDT (PDB code 6QAI) through the utilization of AlphaFold 1 [40]. Then, we compute the pK values of the N-terminus by using the H++ protonation predictor program (http://biophysics.cs.vt.edu/H++) [41], revealing a pKa range of 7.3–7.4 for the N-termini of both chains. These findings indicate the potential feasibility of an oriented covalent immobilization of His-*Lm*PDT through the N-termini at pH 7.5. As a result of the catalyst load experiments (enzyme/support mass ratio), PDT-MIONP3 (1358 IU g^−^^1^; retained activity 36%) was selected as a candidate for further synthetic studies (Table 1).

Lower retained activity values (32.4–36%) were consistently observed for PDT-MIONP derivatives across all cases, as evidenced in Table 1. These findings align with those reported for the covalent immobilization of dimeric PDT from *Trypanosoma brucei*, *Tb*PDT (34% retained activity) [32]. Structural analysis of both dimeric enzymes, *Tb*PDT (PDB ID 2A0K) and *Lm*PDT (PDB ID 6QAI), reveals that the N-termini residues from both chains are located within the same plane. Since both N-termini are involved in the covalent attachment to the support, such spatial arrangement may potentially hamper substrate accessibility to the active site pocket, consequently leading to a notable decrease in enzyme activity. Additionally, the immobilization process might have perturbed the oligomeric assembly of the enzyme and the active site architecture, thereby resulting in diminished activity. Considering the fundamental role of dimer formation for *Lm*PDT functionality [31,42,43], this disruption could further contribute to the observed reduction in activity. Another plausible explanation for this activity loss could be the incomplete attachment of all subunits to the support, thereby increasing the likelihood of subunit dissociation. Additionally, covalent immobilization may introduce constraints on the protein’s flexibility, thereby impacting its catalytic activity.

### 3.2. Substrate Specificity of PDT-MIONP Derivatives

**Figure 2:**
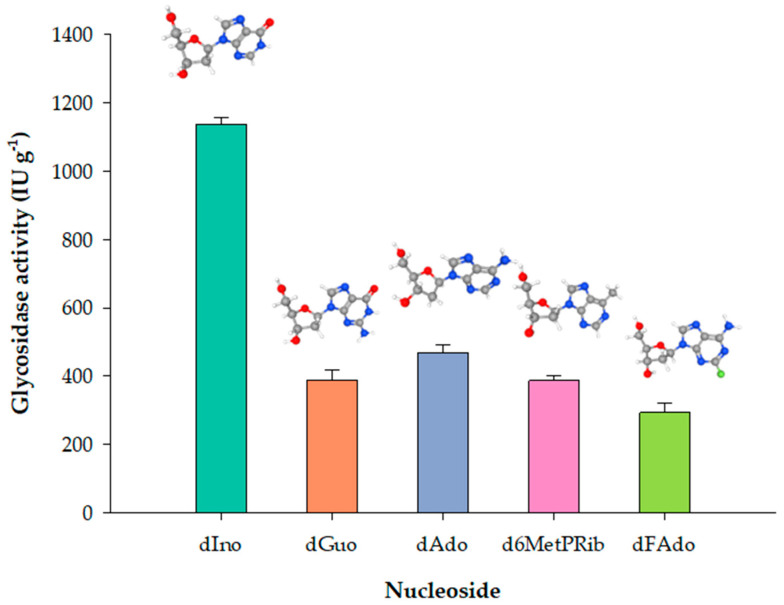
Substrate specificity of MIONPs over different natural and non-natural nucleosides.

The substrate specificity of PDT-MIONP_3_ was assessed by studying the glycosidase activity over a series of natural and non-natural 2′-deoxynucleosides. Similar to the soluble enzyme [29], PDT-MIONP3 exhibited a remarkable preference for dIno over other substrates among the natural 2′-deoxynucleosides (Figure 2) (Figure S1). Interestingly, PDT-MIONP3 also exhibited significant glycosidase activity toward nucleoside analogs FdAdo and d6MetPRib, well-known potential prodrugs used in DEPT strategies [3,6,17,18,19]. As described in the literature, the C-N cleavage of these nucleoside prodrugs releases 2-fluoroadenine (2-FAde) and 6-methylpurine (6-MetPur), respectively, which are typical antimetabolites used in cancer treatment.

### 3.3. Nanoparticles Characterization

**Figure 3:**
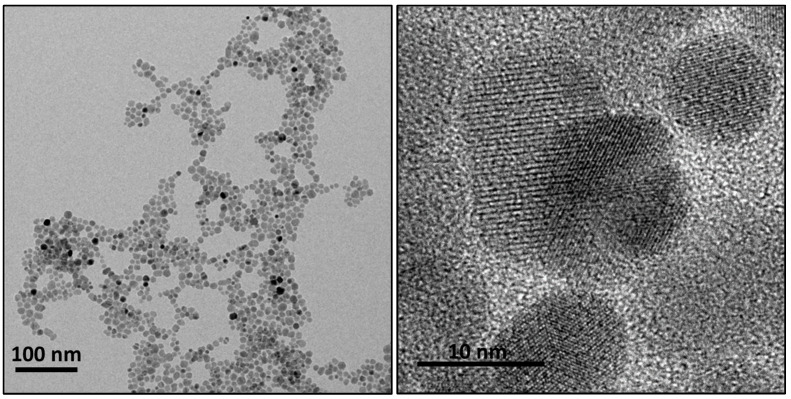
TEM images of a MIONP suspension at 100–10 nm scale.

The size and shape of nanoparticles directly influence immobilization yield and, consequently, the activity of derivatives, as well as the effect of carriers on cell viability, their magnetic properties, organ internalization, and renal clearance [44]. Therefore, the size of carriers used for enzyme immobilization (MIONPs) was previously analyzed by TEM. TEM images (Figure 3) revealed a homogeneous spherical shape with an approximate diameter of 10 nm, which is smaller compared to previously described magnetic nanocarriers [45,46]. Previous studies have shown that stronger protein–particle interactions occur with larger nanoparticles, resulting in more protein unfolding and decreased enzymatic activity [47]. Considering this, the obtained surface area-to-volume ratio was notably high, allowing an increased reactivity and higher immobilization yields. Hence, these MIONPs were considered an ideal alternative as enzymatic carriers, as they were anticipated to exhibit potentially high enzymatic activity, enabling high levels of prodrug activation using fewer of these nanosystems. Additionally, the smaller size of these carriers may favor renal clearance, facilitating their elimination after treatment [44]. 

Moreover, DLS analysis of the stored MIONPs in Milli-Q^®^ water revealed their instability in this solvent, leading to aggregation during storage and resulting in large structures with a hydrodynamic size of 56.64 nm and 4.48 nm before and after sonication, respectively. Furthermore, enzyme immobilization also contributed to an enlargement of the NDT-MIONPs derivatives, with an approximate size of 353.86 nm in Milli-Q^®^ water. This phenomenon should be taken into account when evaluating nanoparticle clearance, as this enlargement may hinder their removal, leading to local accumulation in vivo [44]. However, this issue can be addressed by coating the surface of nanoparticles with different polymers [48].

Conversely, size measurements of MIONPs and NDT-MIONPs conducted without sonication and when diluted in culture media via single particle ICP-MS indicated a mean size of 66.5 nm for MIONPs and 86.5 nm for PDT-MIONPs, showcasing a marginal increase in size. This augmentation is likely attributable to His-*Lm*PDT immobilization, nanoparticle aggregation in solution, and potential interactions between MIONPs and serum components [49]. This size increase could influence their cellular internalization, as demonstrated later in the TEM images of the intracellular uptake of MIONPs and PDT-MIONPs. Comparable investigations involving magnetic nanoparticles [50] revealed that the dynamic corona formed around such nanoparticles minimally affects particle size or cytocompatibility of nanocarriers. However, discernible alterations in zeta potential, magnetization saturation, cellular uptake, and hyperthermia properties were observed.

The zeta potential (z-potential) measurements conducted without prior sonication revealed a significant disparity between MIONP and PDT-MIONP derivatives. While the former exhibited a z-potential of −47.9 mV, consistent with descriptions of other carriers [50], the latter demonstrated a zeta potential of −78.2 mV. Despite both nanocarriers maintaining a negative surface charge, the immobilization process appears to augment this negative value. This elevation could be attributed to the presence of His-*Lm*PDT on the surface of MIONPs, with a predicted total charge of −8 at pH 7 according to the H++ web server. Previous studies have explored the impact of nanoparticle surface charge on cellular interactions, revealing that the negative surface charge of MIONPs correlates with reduced cytotoxicity and cellular uptake compared to cationic carriers [51]. Interestingly, as further described, our findings indicate the efficient uptake of PDT-MIONPs by HeLa and NIH3T3 cells, despite the higher negative zeta potential value.

Moreover, the use of a magnetic matrix as an immobilization support may also provide many other interesting advantages for in vivo use, such as: (i) targeted delivery (magnetic supports can be directed to specific sites within the body using an external magnetic field, which allows for precise delivery of the enzyme to the tumor site, minimizing the impact on healthy tissues), or (ii) applications in hyperthermia (magnetic nanoparticles can be used in hyperthermia treatment, where they are subjected to an alternating magnetic field to generate localized heat) [9,10]. These properties collectively enhance the potential efficacy of the treatment while reducing side effects and systemic toxicity.

### 3.4. Cytotoxicity Analysis of MIONPs and NDT-MIONPs

Although the ultimate IDEPT strategy involves coating MIONPs with specific biological compounds to enhance their biocompatibility and stability, this coating may be compromised during therapy delivery or nanoparticle internalization, thereby exposing the uncoated iron oxide core. Superior biocompatibility and low cytotoxicity render magnetic nanoparticles promising candidates in cancer therapeutics [52]. Therefore, investigating the impact of uncoated carriers on cell viability is crucial to ensure safe IDEPT administration. In this study, we evaluated the effect of MIONPs on mitochondrial activity.

**Figure 4:**
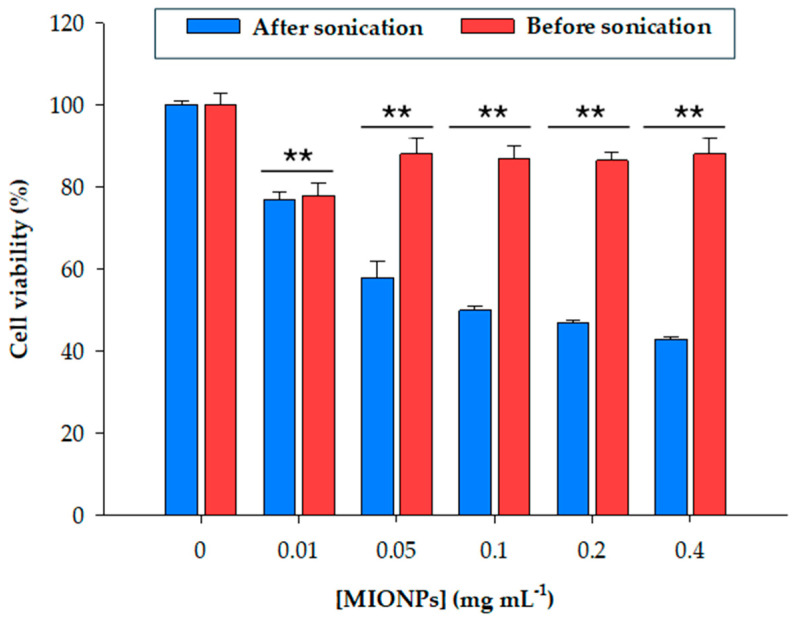
Relative viability of HeLa cells after 24 h of incubation with different concentrations of sonicated and non-sonicated MIONPs (0.01–0.4 mg mL^−^^1^). ** indicate significant differences from controls (*p* < 0.001).

Cell viability after incubation with different concentrations of MIONPs was also studied through the evaluation of mitochondrial activity by MTT assay. As depicted in Figure 4, cell viability exhibited a gradual decrease with increasing nanoparticle concentration.

Given that nanoparticle aggregation resulting from storage significantly influenced carrier size, cell viability was evaluated both with and without nanoparticle sonication. As illustrated in Figure 4, MTT assay results indicated a dose-dependent reduction in mitochondrial activity upon exposure to sonicated MIONPs. After 24 h of incubation, cell viability decreased to 74% at the lowest nanoparticle concentration (0.01 mg mL^−^^1^), with an observed IC_50_ of 0.1 mg mL^−^^1^, representing the nanoparticle concentration inhibiting 50% of the cell population. Beyond this concentration, viability percentages remained relatively stable despite an increase in nanoparticle concentration.

Notably, the assessment of mitochondrial activity after 24 h of incubation with non-sonicated MIONPs revealed that nanoparticle aggregation facilitated higher and more consistent viability values at higher derivative concentrations. Specifically, cell viability remained at approximately 85% even at a concentration of 0.4 mg mL^−^^1^ for non-sonicated MIONPs, while the same concentration resulted in 45% cell survival for sonicated MIONPs. After 30 min of sonication, MIONPs likely approached their original size as observed under TEM, whereas non-sonicated nanoparticles constituted structures 400 times larger. This insight was critical in establishing the handling protocol for these nanoparticles in the immobilization technique and for future applications.

These findings align with previously reported data [46], which revealed a dose-dependent decline in cell viability when investigating the mitochondrial activity of HeLa cells following 24 h of incubation with various concentrations of MIONPs. Nonetheless, the viability values obtained in their experiments were notably higher than those observed in our study. For instance, at the highest concentration assessed (0.4 mg mL^−^^1^), more than 70% of mitochondrial activity was retained [46]. Similar observations were reported in other studies [53,54].

In addition, to provide more insights into the possible mechanisms leading the cytotoxic effect of uncoated magnetic nanoparticles on HeLa cells, we conducted a preliminary Annexin V and propidium iodide-based assay to detect apoptosis (Figure S4; Supplemental Material). Results indicated that the exposure to both concentrations (0.05 mg/mL and 0.1 mg/mL) of MIONPs as well as to 2.5 µM of dF-Ado and F-ade had a noticeable effect on apoptosis, as we detected only a little presence of both early and late apoptotic cells (−5% and 10% in cells exposed to the treatments). This slight discrepancy with the viability assay may suggest the existence of other concomitant mechanisms beyond apoptosis by which such nanoparticles affect cells viability [3].

**Figure 5:**
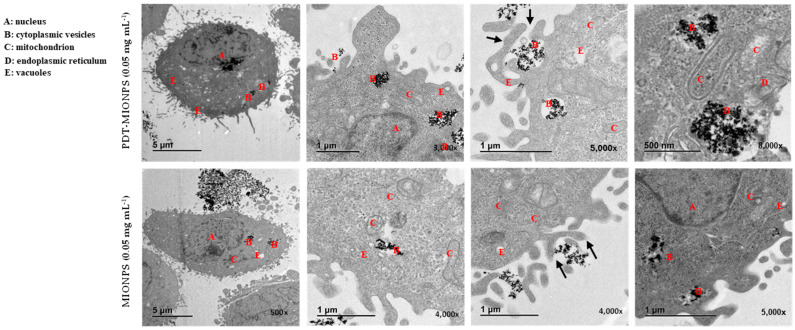
TEM images of the intracellular uptake of MIONPs and PDT-MIONPs by HeLa cells after 24 h of exposure. Black arrows denote the interaction between MIONPs and the microvilli on the HeLa cell surface, while black circles highlight the presence of magnetic nanoparticles. The images are presented from low to high magnification sequences, detailing the local areas within the HeLa cells.

**Figure 6:**
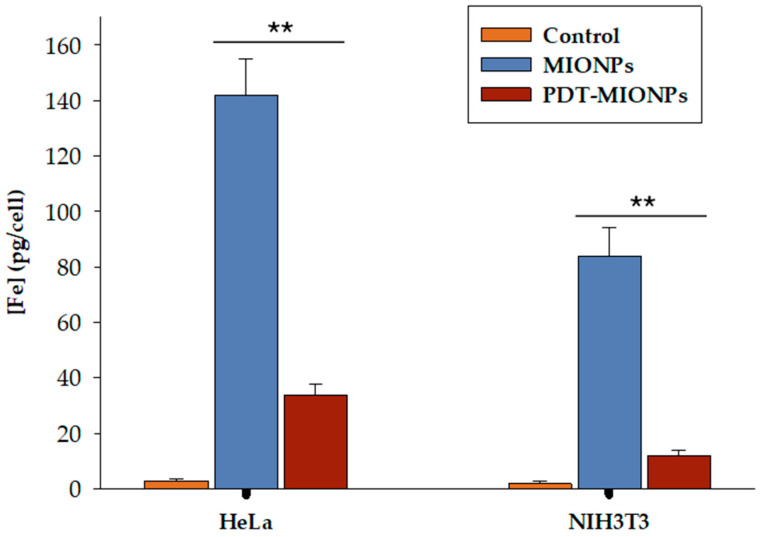
Total iron concentration in HeLa and NIH3T3 cells after 24 h of incubation determined by ICP-MS. ** indicate significant differences from controls (*p* < 0.001).

Moreover, as further described in the intracellular uptake experiments (Figure 5 and Figure 6), our findings indicate a clear direct relationship between nanoparticle size, intracellular accumulation, and cell viability (Figure 4). This is particularly noteworthy considering that our MIONPs were initially smaller than those studied in previous works. Thus, smaller nanoparticles may enter cells more easily, and their accumulation within cells, coupled with the absence of in vivo clearance in cell culture techniques, could contribute to increased cytotoxicity. Similar studies have also reported a direct relationship between carrier size and cytotoxicity, albeit observed for size differences of up to 1000-fold [55]. Furthermore, the literature suggests that the relationship between nanoparticle size and cell uptake may not be linear and is potentially dependent on cell type and cellular interactions [56,57]. However, our TEM images with cells (Figure 5) and ICP-MS results (Figure 6) demonstrated efficient uptake of non-sonicated MIONPs and PDT-MIONPs.

Furthermore, given that enzyme immobilization on nanoparticles increases the size of derivatives and may consequently affect their interaction with cells, the impact on cell viability of PDT-MIONPs was also assessed. The results indicated a mean cell viability of 66% (Figure S3). While one might anticipate higher viability following exposure to large-sized PDT-MIONP derivatives, these findings revealed an intermediate value between sonicated and non-sonicated nanoparticles. This slight reduction in mitochondrial activity compared to non-sonicated MIONPs may be attributed to reduced intracellular accumulation, likely due to their larger size. Conversely, the increased cell viability observed after treatment with PDT-MIONPs compared to sonicated MIONPs could be attributed to the enhanced interaction of PDT-MIONPs with the cell surface, possibly facilitated by the presence of the enzyme on their surface. As earlier mentioned, this hypothesis is supported by the results of the ICP-MS study (Figure 6), which compares the cellular uptake of non-sonicated MIONPs and PDT-MIONPs. Finally, our MIONPs exhibit high viability percentages compared to other described magnetic nanocarriers. For instance, ZnO and CrO_3_ nanoparticles induce a complete loss of mitochondrial activity in Neuro-2A cells at concentrations as low as 0.2 mg mL^−^^1^ [57].

### 3.5. Intracellular Uptake of MIONPs and PDT-MIONP

A common challenge in achieving the effective utilization of magnetic nanoparticles for various biomedical applications such as MRI, tissue repair, targeted drug delivery, and hyperthermia is ensuring sufficient uptake of these nanosystems by specific cells [58]. To address this challenge, it is crucial to consider factors such as nonspecific targeting and low efficiency of internalization [59].

The intracellular uptake of MIONPs and PDT-MIONPs by HeLa cells was examined qualitatively using TEM. The images obtained (Figure 5) showed efficient internalization of aggregates from both nanosystems within the cells. Notably, this qualitative assessment suggested a higher uptake of MIONPs without the enzyme component, potentially explaining the reduced viability observed with PDT-MIONPs, as discussed earlier. Subsequent analysis with ICP-MS (Figure 6) further supported this observation, consistent with findings from previous studies utilizing similar types of MIONPs and employing ICP-MS for the quantification of internalized nanoparticles [33,34,60]. Additionally, a lower level of internalization was observed in NIH3T3 cells compared to HeLa cells. This finding aligns with the previous literature indicating that HeLa cells exhibit greater permissiveness to nanoparticle internalization compared to other non-cancerous cell types This difference can be attributed to the expression of specific cell surface receptors, differences in endocytic pathways, cellular membrane properties, and inherent cellular physiological characteristics [61,62].

The TEM images show an accumulation of aggregated magnetic nanoparticles in cytoplasmic vesicles of HeLa cells after 24 h exposure to the MIONPs and PDT-MIONPs. None of the pictures obtained revealed the presence of such nanoparticles in the nucleus. These images also show the attachment of MIONPs and PDT-MIONPs on the cell surface and the intertwining between the cell microvilli and the MIONPs, which are consistent with the results obtained by Mak et al. using uncoated magnetic nanoparticles [46]. These findings also corroborate those of Calero et al. [60], who investigated similar nanoparticles in size and composition at a concentration of 0.5 mg mL^−^^1^ on HeLa cells. Their study demonstrated the internalization of these nanoparticles via an energy-dependent process, such as endocytosis. As Li et al. [46] demonstrated, this uptake mechanism of MIONPs can result in different cellular responses varying the amount of nanoparticles, time exposure, and cell type.

### 3.6. Application of PDT-MIONP/Prodrug System on Cancer Cells

In previous work, we demonstrated that the prodrug dFAdo remains non-toxic over a broad range of low concentrations (0.5–5 µM), only significantly affecting cell viability when higher concentrations are tested (50 µM). In contrast, the active drug (2-FAde) exhibited a 40–70% inhibition at low concentrations (0.5–1 µM), with an IC_50_ value approximately around 0.65 µM. These results illustrated the unharmful effect of the dFAdo prodrug [6].

Drawing from these findings, we conducted an assessment of the stability and behavior of immobilized derivatives over a 24 h reaction period at 37 °C, before the application of the PDT-MIONP/Prodrug system on cancer cells. Experimental results revealed that the PDT-MIONP derivative successfully converted 83.1% of dFAdo into 2-FAde during the evaluated time frame, demonstrating the enduring catalytic capacity of the PDT-MIONP derivative under physiological conditions over extended durations.

**Figure 7:**
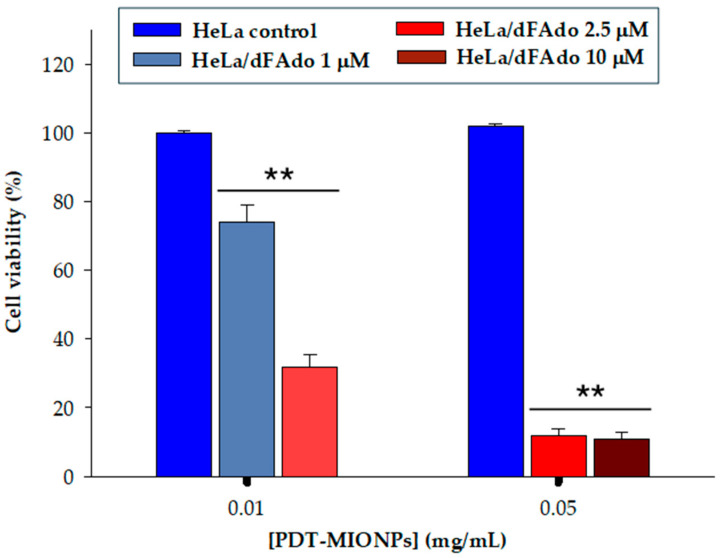
Relative cell viability (%) of HeLa cells 24 h of incubation with different concentrations of PDT-MIONP/prodrug system. ** indicate significant differences from controls (*p* < 0.001).

In the final stage, as an initial exploration of the proposed IDEPT strategy, the developed PDT-MIONP/prodrug system underwent testing on HeLa cells using the MTT assay. This evaluation considered previous cytotoxicity findings and involved combining various concentrations of PDT-MIONPs and dFAdo. The results revealed heightened cytotoxicity when both PDT-MIONPs and prodrug concentrations were increased. Specifically, cell viability decreased to 74% and 32% following treatment with 0.01 mg ml^−^^1^ PDT-MIONPs combined with 1 µM or 2.5 µM of prodrug, respectively (Figure 7). As expected, further reductions in cell viability were observed with the addition of 0.05 mg mL^−^^1^ of MIONPs and 2.5 µM or 10 µM prodrug, resulting in 12% and 11% cell survival, respectively. Thus, the notable decrease in cell viability primarily stemmed from the action of the active compound generated by the immobilized enzyme from the provided prodrug.

These results parallel those reported by Acosta et al. [6] in the sole NDT-based DEPT strategy documented in the literature. While their *Lactobacillus delbrueckii* NDT/prodrug GEDPT system induced slightly higher cytotoxicity in HeLa cells (with 2.64% cell viability observed for 10 µM of prodrug), this strategy may encounter challenges related to poor gene transduction efficiencies and low expression levels when applied to animal models. These limitations can be addressed by directly delivering the active enzyme through IDEPT strategies, such as the PDT-MIONPs system proposed here.

Furthermore, we observed a comparable cytotoxicity effect (12% cell viability) in tumor cells treated with the free drug (2-FAde 2.5 µM) and those treated with the PDT-prodrug system (PDT-MIONPs 0.05 mg/mL-FdAdo 2.5 µM). This outcome underscores the potential of the designed nanosystems to enhance therapy selectivity, particularly following anticipated active targeting, while reducing the need for undesired excipients in drug delivery, thus achieving comparable toxicity outcomes.

Based on these results, we hypothesize that the decrease in cell viability is mainly caused by the cytotoxic effect of 2-FAde rather than by the direct effect of the nanoparticles themselves. However, the possibility of inducing some common side effects associated with these inorganic nanoparticles, such as the generation of reactive oxygen species (ROS), should not be discarded. The potential ROS formation is particularly significant because ROS can lead to oxidative stress, inflammation, and cell damage, which can contribute to the desirable tumor cell death. This dual effect undoubtedly may enhance the therapeutic effect of the NDT-based DEPT strategy, offering a promising direction for future research and development.

In addition to these findings, another important consideration in evaluating the potential application of these NDT-based DEPT strategies is enzyme promiscuity. While His-*Lm*PDT, a type I NDT, exhibits high specificity for purines, *Ld*NDT, a type II NDT, can act on both purine and/or pyrimidine nucleosides. Consequently, the latter may apply to producing a purine/pyrimidine drug cocktail. In ongoing work, we are developing an *Ld*NDT-MIONP/purine-pyrimidine prodrug system using dFAdo and 2′-deoxy-5-fluorouridine to the generation of a purine/pyrimidine drug cocktail. Moreover, considering the results obtained in this study, further investigations are warranted to (a) ensure enhanced in vitro stability and minimize anticipated in vivo clearance by applying a polymeric coating around the particles; (b) explore the potential of these nanoparticles for tumor cell eradication through additional strategies such as hyperthermia; and (c) develop an active-targeting approach to enhance tumor cell internalization efficiency, thereby facilitating a more selective therapy.

## 4. Conclusions

Herein, we present, for the first time, an immobilized-directed enzyme prodrug therapy (IDEPT) based on the application of immobilized His-*Lm*PDT on MIONPs (PDT-MIONPs) for prodrug activation. Notably, PDT-MIONP demonstrates activity across a wide range of natural and non-natural nucleosides, expanding its applicability beyond dFAdo to include other established purine prodrugs (e.g., 6-methyl-2′-deoxyadenosine) [3].

Interestingly, both MIONPs and PDT-MIONPs exhibited efficient internalization by tumor cells (HeLa), surpassing the internalization observed in non-tumor cells (NIH3T3). Additionally, cell viability studies conducted on tumor cells (HeLa) revealed that PDT-MIONPs exhibited a remarkable prodrug-activating ability, leading to a significant decrease in tumoral cell survival. This marks the first IDEPT strategy based on NDTs reported to date, offering a promising avenue for cancer treatment.

## Figures and Tables

**Table 1:** Effect of enzyme/support mass ratio on the immobilization of His-*Lm*PDT onto MIONPs.

Derivative	Added (mg*_enz_* g_sup_^−1^)	Immobilization Yield (%) ^a^	Biocatalyst Load (mg*_enz_* g_sup_^−1^)	Activity (IU g_sup_^−1^) ^b^	Recovery (%) ^c^
PDT-MIONP1	245	100	245	1358 ± 25	36.0
PDT-MIONP2	1035	31	327	1690 ± 50	33.6
PDT-MIONP3	1417	27	395	1970 ± 54	32.4

^a^ Immobilization yield: (initial amount of enzyme/final amount of enzyme) × 100. Protein quantification was performed by densitometry using a protein standard curve. ^b^ Reaction conditions: 0.6 μg of immobilized His-*Lm*PDT, [dIno] = 1 mM, in PBS buffer (1×, pH 7.4)., at 37 °C, 5 min, 300 r.p.m. Vr = 80 μL. ^c^ Retained activity (%): (activity derivative/activity His-*Lm*PDT) × 100.

## Data Availability

All data related to this manuscript are available upon request.

## Supplementary Materials

The following supporting information can be downloaded at https://www.mdpi.com/article/10.3390/biom14080894/s1. Figure S1: Chemical structures of all nucleosides and nucleobases used in this work. Figure S2: SDS-PAGE analysis of soluble His-*Lm*PDT. Figure S3: Determination of relative viability of HeLa cells at different concentrations of PDT-MIONPs (0.01–0.1 mg mL^−1^). Figure S4. Dot plot showing the results of cell apoptosis assay using Annexin V and propidium iodide on Hela cells after 24 h of exposure to different concentrations of MIONPs: (A) 0.1 mg/mL or (B) 0.05 mg/mL, (C) dF-Ado (2.5 µM) and (D) F-Ade (2.5 µM). The early apoptotic cells are located in Q3 and the late apoptotic or necrotic cells are included in Q2.
