# Supplementary material for: Novel Directed Enzyme Prodrug Therapy for Cancer Treatment Based on 2′-Deoxyribosyltransferase-Conjugated Magnetic Nanoparticles
Source: Biomolecules. 2024 Jul 24;14(8):894. doi: 10.3390/biom14080894 (PMC11352528; doi:10.3390/biom14080894)
Supplement: Supplementary file 1 [file biomolecules-14-00894-s001.zip › biomolecules-3063442-supplementary.pdf]

biomolecules

Article

Novel directed enzyme prodrug therapy for cancer treatment based on 2'-deoxyribosyltransferase-conjugated magnetic nanoparticles.

Elena Pérez [1,†], Javier Acosta [1,†], Victor Pisabarro [1], Marco Cordani [2,3], José C. S. dos Santos [4], Jon Sanz-Landaluze [5], Juan Gallo [6], Manuel Bañobre-López [6] and Jesús Fernández-Lucas [1,3,7,*]

[1] Applied Biotechnology Group, Universidad Europea de Madrid, Urbanización El Bosque, 28670 Villaviciosa de Odón, Spain; elena.perez2@universidadeuropea.es (E.P.), javier.acosta@universidadeuropea.es (J.A.), victorpisabarromontoro@gmail.com (V.P.)
[2] Instituto de Investigaciones Sanitarias San Carlos (IdISSC), 28040 Madrid, Spain
[3] Department of Biochemistry and Molecular Biology, Faculty of Biology, Universidad Complutense de Madrid, C. de José Antonio Novais, 12, 28040 Madrid, Spain; mcordani@ucm.es
[4] Instituto de Engenharias e Desenvolvimento Sustentável, Universidade da Integração Internacional da Lusofonia Afro-Brasileira, Campus das Auroras, Redenção 62790970, CE, Brazil; jcs@unilab.edu.br
[5] Department of Analytical Chemistry, Faculty of Chemical Science, Universidad Complutense de Madrid, Avenida Complutense S/N, 28040 Madrid, Spain; jsanzlan@quim.ucm.es
[6] INL—International Iberian Nanotechnology Laboratory, Avenida Mestre José Veiga, 4715-330 Braga, Portugal; juan.gallo@inl.int (J.G.), manuel.banobre@inl.int (M.B.-L.)
[7] Grupo de Investigación en Ciencias Naturales y Exactas—GICNEX, Universidad de la Costa, CUC, Calle 58 # 55–66, 080002 Barranquilla, Colombia
* Correspondence: jesusf08@ucm.es
† These authors contributed equally to this work.

Supplemental material

Figures

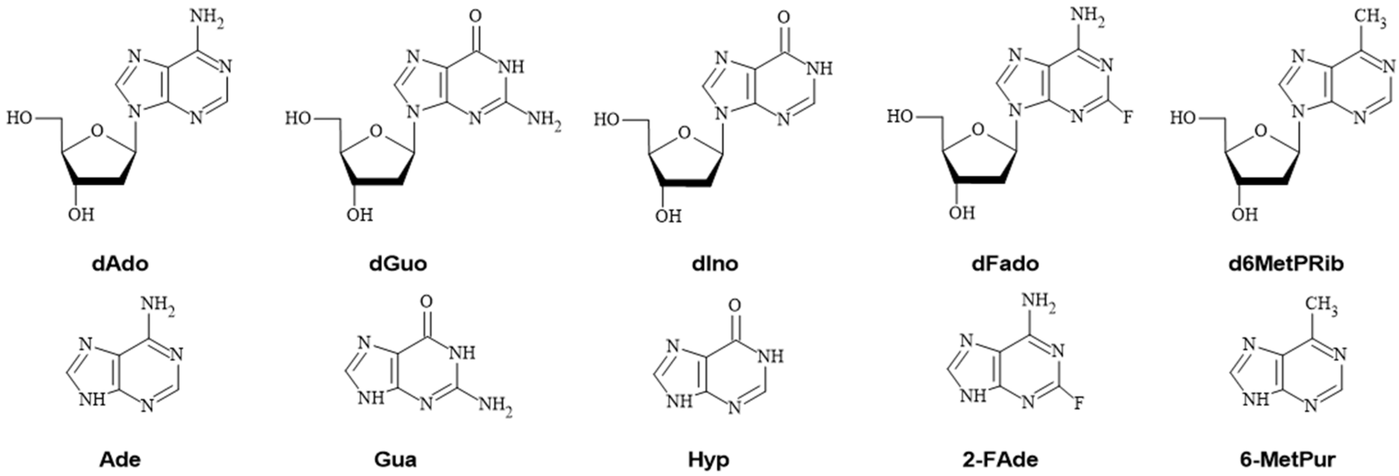

Figure S1. Chemical structures of all nucleosides and nucleobases used in this work.

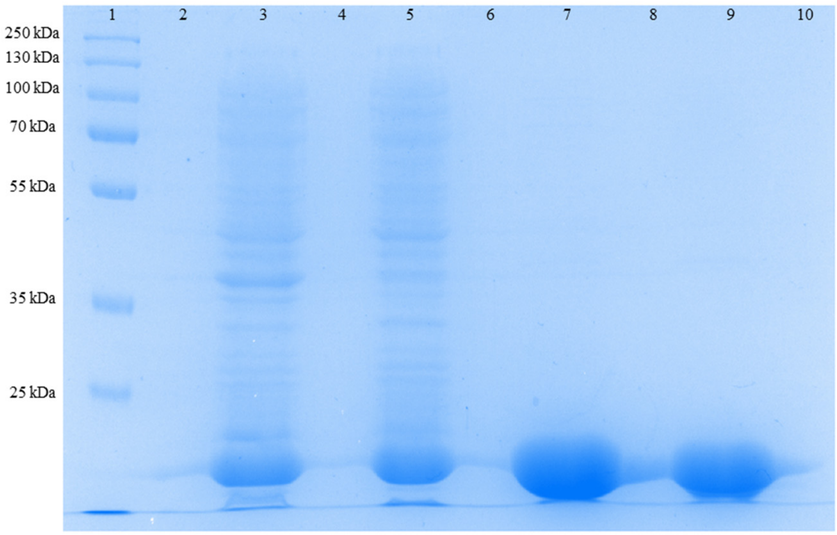

Figure S2. SDS-PAGE analysis of soluble His-*Lm*PDT. Lane 1 contains PageRuler Plus Prestained Protein Ladder (Thermo Scientific ™); lane 3, cell lysate extract; lane 5, soluble lysate free of cell bodies; lane 7, purified and concentrated eluted fractions; lane 9, positive control of purified His-*Lm*PDT.

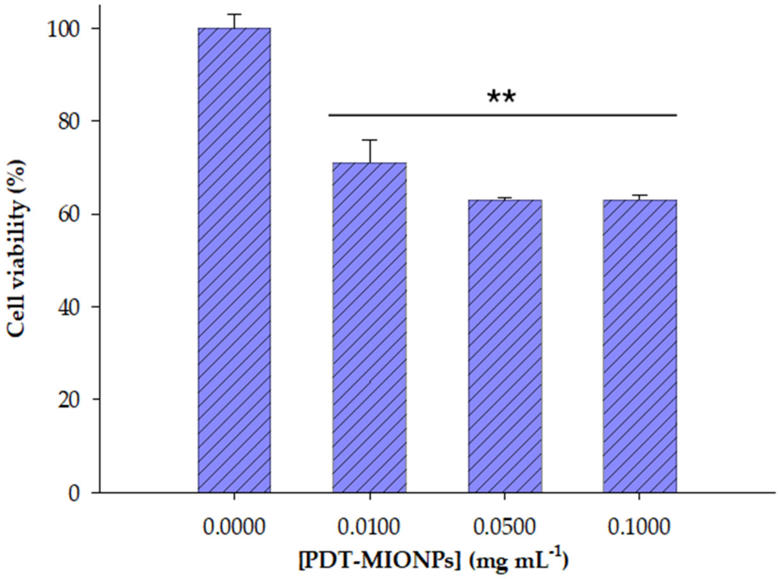

Figure S3. Determination of relative viability of HeLa cells at different concentrations of PDT-MIONPs (0.01-0.1 mg mL^{-1}, 24 h). **a** indicates significant differences from controls (p<0.001)

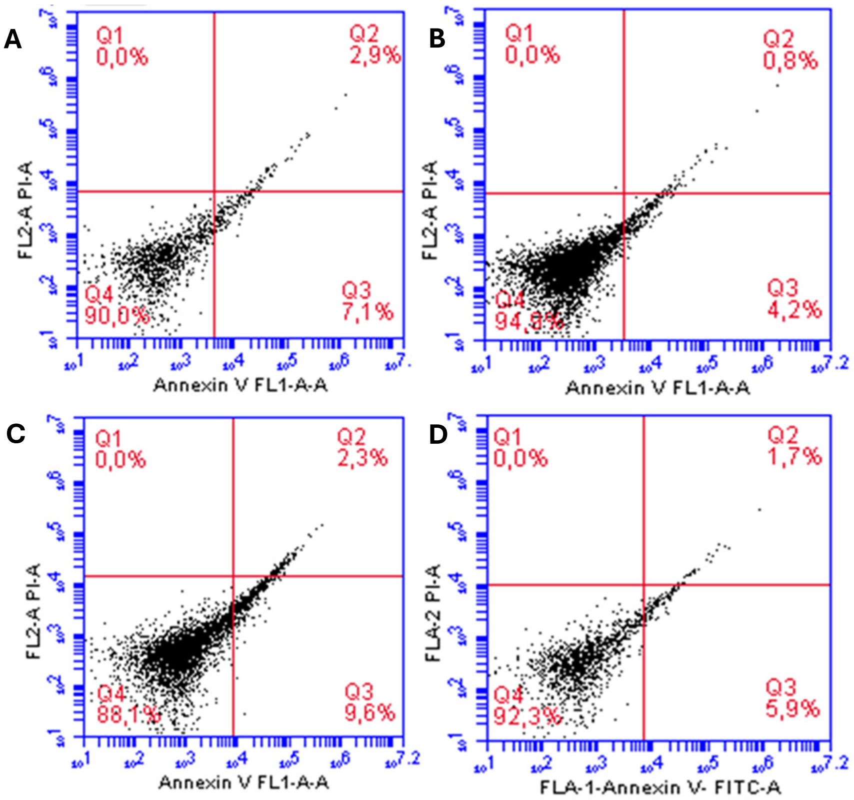

Figure S4. Dot plot showing the results of cell apoptosis assay using Annexin V and propidium iodide on Hela cells after 24h of exposure to different concentrations of MIONPs: A) 0,1 mg/ml or B) 0,05 mg/ml, C) dF-Ado (2,5 μM) and D) F-ade (2,5 μM). The early apoptotic cells are located in Q3 and the late apoptotic or necrotic cells are included in Q2.
